# Urinary Transforming Growth Factor-beta 1 as a marker of response to immunosuppressive treatment, in patients with crescentic nephritis

**DOI:** 10.1186/1471-2369-6-16

**Published:** 2005-12-20

**Authors:** Dimitrios S Goumenos, Pantelitsa Kalliakmani, Sotiris Tsakas, Florentia Sotsiou, John G Vlachojannis

**Affiliations:** 1 Department of Internal Medicine-Nephrology, University Hospital, Patras, Greece; 2 Department of Biology, University of Patras, Patras, Greece; 3 Department of Pathology, General Hospital "Evangelismos", Athens, Greece

## Abstract

**Background:**

Crescentic nephritis is characterized by formation of cellular crescents that soon become fibrotic and result in irreversible damage, unless an effective immunosuppressive therapy is rapidly commenced. TGF-β_1 _is involved in the development of crescents through various pathways. The aim of this study was to identify whether the determination of urinary TGF-β_1 _levels in patients with crescentic nephritis could be used as a marker of response to treatment.

**Methods:**

Fifteen patients with crescentic nephritis were included in the study. The renal expression of TGF-β_1 _was estimated in biopsy sections by immunohistochemistry and urinary TGF-β_1 _levels were determined by quantitative sandwich enzyme immunoassay (EIA). TGF-β_1 _levels were determined at the time of renal biopsy, before the initiation of immunosuppressive treatment (corticosteroids, cyclophosphamide and plasma exchange). Twelve patients with other types of proliferative glomerulonephritis and ten healthy subjects were used as controls.

**Results:**

Improvement of renal function with immunosuppressive therapy was observed in 6 and stabilization in 4 patients (serum creatinine from 3.2 ± 1.5 to 1.4 ± 0.1 mg/dl and from 4.4 ± 1.2 to 4.1 ± 0.6 mg/dl, respectively). In 5 patients, with severe impairment of renal function who started on dialysis, no improvement was noted. The main histological feature differentiating these 5 patients from others with improved or stabilized renal function was the **percentage **patients with poor response to treatment were the percentage of glomeruli with crescents and the presence of ruptured Bowman's capsule and glomerular necrosis. Urinary TGF-β_1 _levels were significantly higher in patients who showed no improvement of renal function with immunosuppressive therapy (930 ± 126 ng/24 h vs. 376 ± 84 ng/24 h, p < 0.01). TGF-β_1 _was identified in crescents and tubular epithelial cells, whereas a significant correlation of TGF-β_1 _immunostaining with the presence of fibrocellular cresents was observed (r = 0.531, p < 0,05).

**Conclusion:**

Increased TGF-β_1 _renal expression and urinary excretion that is related to the response to immunosuppressive therapy was observed in patients with crescentic nephritis. Evaluation of urinary TGF-β_1 _levels may be proved a useful marker of clinical outcome in patients with crescentic nephritis.

## Background

Crescentic nephritis is a type of glomerular disease characterized by crescent formation followed by a rapidly progressive course [1]. It can occur in cases with antibodies against glomerular basement membrane (anti-GBM disease), secondary to other glomerulonephritis and in patients with vasculitis and presence of antineutrophilic cytoplasmic antibodies (ANCA) [1]. Proliferation of parietal cells of Bowman's capsule and macrophages stimulated by cytokines and growth factors is implicated in the development of cellular crescents that soon become fibrotic and result in irreversible damage [2].

TGF-β represents a group of 25-kD proteins that are actively involved in the development and differentiation of various tissues and in the healing process after a tissue injury [3]. Three isoforms of TGF-β have been identified in mammalian species and TGF-β_1 _is the most commonly found in humans [3]. Normally, TGF-β_1 _release ceases by feedback mechanisms when the healing process has been completed [3,4]. However, if TGF-β_1 _release is not switched off, extracellular matrix components (ECM) are accumulated and tissue fibrosis occurs [4]. TGF-β_1 _is involved in the development of scarring in crescentic nephritis via activation of myofibroblasts from glomerular parietal epithelial cells [5]. Interstitial myofibroblasts also contribute to the development of fibrous crescents through their migration into the Bowman's space of glomeruli with disrupted capsules [6]. The implication of TGF-β_1_is further supported by the observation of amelioration of histologic damage in experimentally induced anti-GBM nephritis with the blockade of TGF-β_1 _action [7].

Increased urinary excretion of TGF-β_1 _has been reported in experimentally induced crescentic nephritis that was related to a scarring process leading to end-stage renal disease [8]. Elevated urinary TGF-β_1 _levels have been observed in patients with crescentic nephritis and IgA nephropathy that were reduced after treatment with corticosteroids [9]. In the present study the renal expression and urinary excretion of TGF-β_1 _were examined in patients with crescentic nephritis in order to identify any potential relation of urinary TGF-β_1 _levels with the response to treatment with corticosteroids and cyclophosphamide.

## Methods

### Patients

Fifteen patients (7M/8F, aged from 23–74 years) with crescentic nephritis due to anti-GBM nephritis (n = 1), diffuse lupus nephritis (n = 1), Henoch Schönlein purpura (n = 2) and ANCA(+) vasculitis (n = 11) were included in the study. The mean serum creatinine and urinary protein at presentation were 4.4 ± 1.8 mg/dl and 2.0 ± 1 g/24 h respectively (Table 1). The expression of TGF-β_1 _in the kidney was estimated by immunohistochemistry in biopsy sections obtained from all patients whereas TGF-β_1 _urinary levels were measured at the time of biopsy and before the administration of immunosuppressive therapy in 12 patients and compared to those observed in 12 patients with other types of proliferative GN (IgA nephropathy n = 7, mesangiocapillary GN n = 2 and endocapillary proliferative GN n = 3) and in 10 healthy subjects.

All patients were treated with methylprednisolone pulse (1 g/day intravenously for 3 days) followed by oral methylprednisolone (0.5 mg/kg BW/d) and cyclophosphamide either orally (2 mg/kg BW/d) or intravenously (0.7 g/m^2^). Plasma exchange (10 sessions of 4 liters each) was also performed in 6 patients. One of these had anti-GBM disease and the other 5 had signs of uremia and needed hemodialysis at presentation. All patients were followed up for at least 3 months after the initiation of therapy. At that time a second measurement of urinary TGF-β_1 _levels was performed in most patients with improved or stabilized renal function without the need of dialysis.

### Conventional histopathology

All renal tissues obtained by needle biopsy (14 G) were fixed in 10% neutral buffered formalin, embedded in paraffin and examined in multiple levels (4 μm). Pathologic examination included hematoxylin and eosin (H&E), periodic acid Schiff (PAS), Masson trichrome and reticulin silver methenamine stain. Crescent organization was evaluated by the presence of reticulin fibers among the cellular components of the crescent. The presence of reticulin allowed identification of fibrocellular and fibrotic crescents. The percentage of the glomeruli surrounded by crescents (cellular, fibrocellular, fibrotic) was counted. Rupture of the Bowman's capsule was also detected on reticulin and PAS sections. The severity of interstitial fibrosis, inflammation and tubular atrophy were also estimated and expressed as mild and severe.

### Immunohistochemistry

TGF-β_1 _was detected immunohistochemically on kidney biopsy sections. Briefly, formalin fixed paraffin-embedded kidney sections (4 μm) were cut and mounted on gelatinized slides. De-waxed and hydrated sections were processed in a microwave oven for 3 cycles, 5 min each, at 450 W in citrate buffer, pH 6.0. After cooling, sections were quenched with H_2_O_2 _3% in methanol for 20 min to inhibit endogenous peroxidase activity. Samples were then treated with Protein Blocking Agent to reduce the non-specific binding of antibodies. Slides were washed in phosphate buffered saline (PBS) and incubated with a polyclonal anti-TGF-β_1 _antibody (1:50) (Santa Cruz, USA) for 1 h at 37°C, in a humid atmosphere. Following antibody treatment, slides were washed in PBS and incubated for 10 min with a polyvalent biotinylated secondary antibody (Kwik kit, IMMUNON™, USA). Slides were then washed in PBS, incubated with streptavidin-peroxidase reagent for 10 min and washed again with PBS. Visualization of the antigen-antibody complex was achieved by incubating slides in diamine-benzidine (DAB) chromogen solution for 10–20 min, until a satisfactory color development. Finally, slides were washed in water, counterstained with hematoxylin for 2 min and mounted with resin mounting medium. All steps, except that of the primary antibody, were performed at RT. Control sections were incubated with non-immune rabbit antiserum or processed after the omission of the primary antibody. The severity of TGF-β_1 _immunostaining was assessed by morphometric analysis and point counting as previously described [10].

### Determination of urinary and plasma TGF-β_1 _levels

The concentration of TGF-β_1 _was measured by EIA, according to Honkanen et al. [11]. Microtitre plates (Costar, USA) were coated with 0.1 μg/well mouse monoclonal anti-TGF-β s antibodies (Genzyme Co., USA) in 0.05 M Na_2_CO_3 _buffer pH 9.0, by incubating overnight at 4°C. The wells were washed with PBS, 0.05% Tween-20. 100 μl of standard dilutions (R&D Systems, UK) and undiluted samples were acid-activated (1 N HCl, for 2 h at RT) and neutralized (1.2 N NaOH / 0.5 M HEPES). 100 μl of neutralized samples were incubated in the wells overnight at 4°C. The TGF-β_1 _bound onto the wells was then detected with a rabbit polyclonal anti-TGF-β_1 _antibody (R&D Systems, UK) labeled with horseradish peroxidase (200 μl, 1.5 h at RT). Peroxidase activity was determined using tetramethylbenzidine (TMB) solution as a substrate (R&D Systems, UK). The intra-assay and inter-assay coefficients of variation (CV) were 7.4 and 6.3%, respectively. The recovery of TGF-β_1 _standards (50 and 100 pg/ml) ranged from 83 to 112%. TGF-β_1 _urinary excretion was calculated in ng/24 h and plasma TGF-β_1 _concentration was calculated in ng/ml.

### Statistical analysis

The results were expressed as means ± SD. Differences of TGF-β_1 _excretion in urine between the patient groups were determined by comparison of their mean and median values, using Student's t-test for unpaired data and Kruskall-Wallis non-parametric test. Linear regression analysis was used for correlation between parameters. Multiple regression analysis was applied to determine the predictive value of a given parameter for the progression of renal failure (final serum creatinine). Any p value <0.05 was considered as significant.

## Results

### General observations/clinical outcome

Six patients (40%) with crescentic GN due to anti-GBM nephritis (n = 1), Henoch-Schönlein purpura (n = 2), lupus nephritis(n = 1) and ANCA(+) vasculitis (n = 2) showed a significant improvement of renal function with immunosuppressive therapy (serum creatinine decreased from 3.2 ± 1.5 to 1.4 ± 0.1 mg/dl). Stabilization of renal function without the need of dialysis was achieved in 4 patients (serum creatinine before and after treatment 4.4 ± 1.2 and 4.1 ± 0.6 mg/dl, respectively).

In 5 patients (33%) with ANCA(+) vasculitis who were presented with signs of uremia no improvement of renal function was observed, even with the addition of plasma exchange in the treatment protocol. The main features differentiating patients who showed improvement of renal function with treatment compared to the others were the serum creatinine at presentation (3.1 ± 1.1 vs. 5.2 ± 1.5 mg/dl, p = 0.03), the percentage of glomeruli with crescents (56% vs. 79%, p < 0.03) and the percentage of patients with ruptured Bowman's capsule and necrosis within the glomeruli (17% vs. 67%, p < 0.05) (Table 2). However, urinary TGF-β_1 _levels were the most significant parameter differentiating patients with response to treatment from the others (930 ± 126 vs. 376 ± 84 ng/24 h, p < 0.01) (Table 2).

A strong predictive value of the intial serum creatinine, percentage of glomeruli with crescents and baseline urinary TGF-β_1 _levels for the final serum creatinine was evident. Among those parameters baseline urinary TGF-β_1 _levels had the strongest predictive value (R^2 ^= 59%, p < 0.01).

### Immunostaining for TGF-β_1_

**Figure 1:**
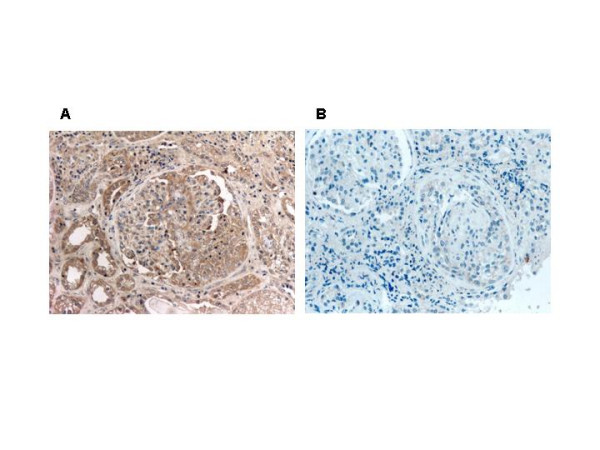
**Immunohistocemical detection of TGF-β_1 _in the renal tissue. **A: Localization of TGF-β_1 _in a crescent and in tubular epithelial cells (dark brown area) (× 200). B: Negative control (× 200).

TGF-β_1 _was localized within the crescents and particularly in areas with cellular and fibrocellular crescents. It was also identified within the cytoplasm of tubular epithelial cells and in small areas of the interstitium (Fig. 1A). A significant correlation of the extent of TGF-β_1 _immunostaining with the presence of fibrocellular crescents was observed (r = 0.531, p < 0.05). However, no relation of the extent and **the intensity **of TGF-β_1 _expression with the response to treatment was identified (Table 2).

### Urinary and plasma TGF-β_1 _levels

**Figure 2:**
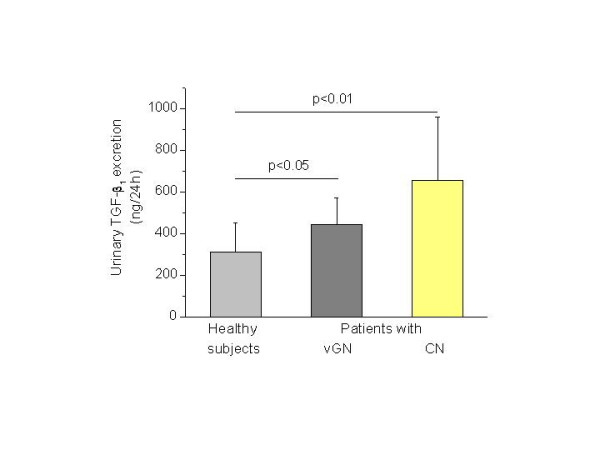
Urinary TGF-β_1 _excretion increased in patients with crescentic nephritis (CN) (n = 15, p < 0.01) and with various types of proliferative GN (vGN) (n = 12, p < 0.05), compared to healthy subjects (n = 10).

**Figure 3:**
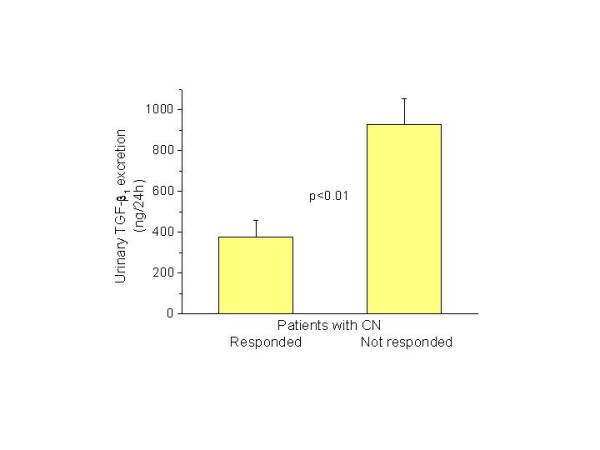
Urinary TGF-β_1 _excretion decreased in patients with crescentic nephritis who responded to immunosuppressive therapy (n = 6) compared to those who showed no improvement of renal function (n = 9).

Elevated urinary TGF-β_1 _levels were identified in patients with crescentic nephritis compared to healthy individuals (653 ± 306 vs. 310 ± 140 ng/24 h) and to those of patients with other types of proliferative GN (653 ± 306 vs. 441 ± 131 ng/24 h, p < 0.05) (Fig. 2). The urinary TGF-β_1 _excretion was significantly lower but still higher than normal in patients with crescentic nephritis who showed improvement of renal function with immunosuppressive treatment compared to those who showed no improvement and had either stabilized renal function or end-stage renal disease after treatment (376 ± 84 vs. 930 ± 126 ng/24 h, p < 0.01) (Fig. 3). No significant alteration in the urinary TGF-β_1_excretion was observed before and after administration of immunosuppressive therapy in patients who showed improvement (352 ± 80 vs. 400 ± 102 ng/24 h, p = NS) or stabilization of renal function (960 ± 81 vs. 963 ± 47 ng/24 h, p = NS).

The plasma TGF-β_1 _levels of patients with crescentic nephritis did not significantly differ compared to those of healthy individuals (31 ± 6 vs. 28 ± 4 ng/ml, p = NS) and were not correlated to the urinary levels.

## Discussion

In this study elevated urinary TGF-β_1 _levels were observed in patients with crescentic nephritis along with TGF-β_1 _expression in cellular and fibrocellular crescents and in the tubulointerstitial area. The excreted amount of TGF-β_1 _in the urine was significantly lower in patients who showed a significant improvement of renal function with immunosuppressive treatment in comparison to those with no signs of improvement and it was positively related to the presence of cellular crescents in the renal biopsy.

Several cytokines and growth factors are involved in the development of crescentic nephritis [2]. Activation and proliferation of epithelial cells, monocytes, macrophages, fibroblasts, myofibroblasts and mast cells have been implicated in the formation of crescents and their evolution to fibrosis [12-14]. TGF-β_1 _is a pluripotent growth factor that is involved in the pathogenesis of experimental and clinical renal scarring [15-17]. It is implicated in the development of fibrous crescents via activation of myofibroblasts from glomerular and tubular epithelial cells [5,18]. In this study TGF-β_1 _was identified within cellular and fibrocellular crescents as well as tubular epithelial cells in the renal tissue of patients with crescentic nephritis. Although the factors involved in organization of cellular to fibrotic crescents have not been fully elucidated, a ruptured Bowman's capsule facilitates the progressive organization of cellular crescents by permitting the entry of activated periglomerular T cells and fibroblasts into Bowman's space [19,20]. Ruptured Bowman's capsule was more frequently observed in the glomeruli of our patients with irreversible damage compared to those with improved or stabilized renal function with treatment. We have previously described correlations between percentage of glomeruli with a disrupted Bowman's capsule, fibrous crescents and expression of interstitial myofibroblasts in patients with crescentic nephritis [21]. It is also known that the migration of interstitial myofibroblasts into the Bowman's space through holes in the Bowman's capsule may also contribute to the pathogenesis of glomerulosclerosis [6]. These cells have been identified within crescents demonstrating features of fibrocellular to fibrous organization [13]. Certain proteoglycans such as versican, biglycan and decorin are also involved in the development of fibrous crescents via activation of myofibroblasts and TGF-β_1 _[22]. Connective tissue growth factor (CTGF) has been recently shown to be involved in the extracellular matrix production in parietal epithelial cells via TGF-β pathway promoting the scarring process in glomerular crescents [23,24].

Increased urinary TGF-β_1 _levels have been reported in patients with membranous nephropathy and other types of glomerular disease characterized by the presence of nephrotic syndrome [10,25]. This has been related to activation of tubular epithelial cells by proteinuria [26]. Increased urinary TGF-β_1 _excretion has been found in patients with insulin-dependent diabetes mellitus and in those with mesangial proliferative glomerulonephritis [27]. Elevated urinary TGF-β_1_activity has been observed in experimentally induced anti-GBM nephritis in rabbits, very early in the course of the disease [8]. Urine TGF-β_1 _activity increased from day 2 onwards peaked on day 7 and returned to normal levels after day 14. This time course was identical to that observed for the renal cortical production of TGF-β_1 _and it was related to the development of fibrotic lesions [8]. These results suggest that measurement of urinary TGF-β_1 _levels at certain stages of the disease may be more useful than serum creatinine in predicting the progression to end stage renal disease and serve as a noninvasive adjunct in monitoring response to therapy [8]. Elevated urinary TGF-β_1 _levels that were correlated to the degree of crescent formation have been described in patients with crescentic nephritis and IgA nephropathy. These levels were significantly reduced after treatment with corticosteroids [9]. In this study, increased urinary TGF-β_1 _levels and TGF-β_1 _renal expression within cellular and fibrocellular crescents were found in patients with crescentic nephritis. These results are in agreement with those observed in experimentally induced anti-GBM nephritis and are suggestive of activation of TGF-β_1 _during an early stage of the disease. At this stage the elevated urinary TGF-β_1 _levels might be due to increased local production from inflammatory cells within the glomeruli. However, at a latter stage during the organization of crescents increased production of TGF-β_1 _from myofibroblasts is possible. It is known that during the development of cellular crescents the administration of immunosuppressive therapy can restrict the glomerular damage whereas the development of fibrotic crescents suggests that permanent damage has occurred. In patients who showed a significant improvement of renal function with immunosuppressive treatment the excreted amount of TGF-β_1 _in the urine was significantly lower compared to that observed in patients with no improvement of renal function. Thus, urinary TGF-β_1 _levels may be a marker of acute glomerular inflammation associated with crescent formation and may represent the severity of glomerular involvement and the potential response to immunosuppressive drugs towards inhibition of the development of permanent damage. According to our results, very high urinary TGF-β_1 _levels are followed by a poor response to immunosuppressive therapy. Furthermore, in patients with poor response to therapy (serum creatinine 4 mg/dl) the urinary TGF-β_1 _levels remain high even after the administration of immunosuppressive therapy. This suggests that very high urinary TGF-β_1 _levels probably represent the development of irreversible damage in the renal tissue of patients with crescentic nephritis. However, since the number of patients examined is not large, further research is required in order to establish urinary TGF-β_1 _levels as a marker of the clinical outcome and response to treatment in patients with crescentic nephritis.

## Conclusion

These findings provide evidence that the determination of urinary TGF-β_1 _levels in patients with crescentic nephritis gives information about the organization of cellular to fibrous crescents and the response to immunosuppressive therapy. However, further research is necessary in order to establish urinary TGF-β_1 _levels as a useful marker of the disease activity and define the exact role of TGF-β_1 _in the progression of crescentic nephritis.

## Competing interests

The author(s) declare that they have no competing interests.

## Authors' contributions

DS Goumenos: Design and supervision of the study

P Kalliakmani: Patients follow up and sample collection

S Tsakas: Immunohistochemical and ELISA experiments

F Sotsiou: Renal biopsies

JG Vlachojannis: Design of the study

## Pre-publication history

The pre-publication history for this paper can be accessed here:



## Figures and Tables

**Table 1:** Clinical and histological features at presentation.

Patients with crescentic nephritis (n = 15)		
Gender (male/female)	7/8	
Age (years)	56 ± 16	
Serum creatinine (mg/dl)	4.4 ± 1.8	
Glomeruli with crescents (%)	70 ± 20	
Cellular crescents (%)	45 ± 30	
Fibrocellular crescents (%)	37 ± 18	
Fibrous crescents (%)	18 ± 17	
Bowman's capsule rupture (# of pts)	Yes (n = 7)	No (n = 8)
Interstitial inflammation (# of pts)	Mild (n = 4)	Severe (n = 11)
Interstitial fibrosis (# of pts)	Mild (n = 6)	Severe (n = 9)

**Table 2:** Clinical and histological features of patients with or without improvement of renal function with immunosuppressive therapy.

	Improvement of renal function with treatment		P
	
	YES (n = 6)	NO (n = 9)	
Serum creatinine (mg/dl)	3.1	5.2	0.03
Urine protein (g/24 h)	2.4	1.7	NS
TGF-β_1 _urine levels (ng/24 h)	376	930	0.001
Glomeruli with crescents (%)	56%	79%	0.03
Cellular crescents (%)	37%	50%	NS
Fibrocellular crescents (%)	44%	31%	NS
Fibrotic crescents (%)	15%	20%	NS
Bowman's capsule rupture and glomerular necrosis [# of pts (%)]	1/6 (17%)	6/9 (67%)	<0.05
Interstitial inflammation (# of pts)	Mild (n = 2) Severe (n = 4)	Mild (n = 2) Severe (n = 7)	NS
Interstitial fibrosis (# of pts)	Mild (n = 5) Severe (n = 1)	Mild (n = 4) Severe (n = 5)	NS
TGF-β_1 _immunostaining (% stained points)	27 ± 8	25 ± 12	NS
